# Transient oligoarthritis of the lower extremity following influenza B virus infection: Case report

**DOI:** 10.1186/1546-0096-8-4

**Published:** 2010-01-14

**Authors:** Normi Bruck, Manfred Gahr, Frank Pessler

**Affiliations:** 1 University Children's Hospital, Medical Faculty "Carl-Gustav-Carus", Technical University Dresden, Germany; 2 Helmholtz Centre for Infection Research, Braunschweig, Germany

## Abstract

A 12-year-old girl developed influenza B virus infection proven by typical symptoms and detection of the virus in a nasopharyngeal swab by culture and PCR. Two weeks later she developed an otherwise unexplained transient oligoarthritis of small joints of the left foot. Influenza viruses may be a hitherto underappreciated cause of a post-infectious arthritis.

## Background

Influenza viruses affect the musculoskeletal system most commonly in the form of myositis, which may range from benign myalgias to life threatening rhabdomyolysis [1]. Although reactive arthritis has been described in adults after influenza immunization [2-4], arthritis associated with influenza virus infection in humans has, to our knowledge, not been documented in the international literature, particularly not in pediatric patients. This is the more surprising since a variety of both RNA and DNA viruses, including parvovirus B19, rubella, hepatitis B and C, Epstein-Barr, varicella zoster and mumps virus, can cause arthritis through direct or indirect mechanisms [5,6]. Here, we report on a 12-year-old Caucasian girl who developed a transient arthritis of a lower extremity after culture- and PCR-proven infection with influenza B virus.

## Case Presentation

In February 2008, a 12-year-old girl developed the flu with high fever, malaise, headaches, photophobia, myalgia and arthralgia. PCR and viral culture of a nasopharyngeal swab demonstrated influenza B virus. Two weeks after the flu symptoms resolved, she complained of painful swelling at the proximal interphalangeal joint of the 3rd left toe and, about one week later, at the left first metatarsophalangeal joint. Physical examination revealed tenderness, erythema, mild swelling, and restricted motion in these joints and in the interphalangeal joint of the left 1^st ^toe. The remaining physical examination was normal. In particular, there was no rash and no enthesitis or enthesalgia. Uveitis was excluded by ophthalmologic examination. Laboratory determinations revealed an elevated eosinophil count (9%) and low serum IgA of 0.45 g/l (normal, 0.70-2.30). Remaining blood counts, C-reactive protein, erythrocyte sedimentation rate, ferritin, liver and kidney parameters, antinuclear antibodies, antistreptolysin titer and rheumatoid factor were normal or negative. Serologic tests were negative for *Borrelia burgdorferi *and *Toxoplasma gondii *and revealed no other acute or recent viral infection including CMV, EBV, adenovirus, parvovirus, mumps, and rubella. A stool culture was not done due to the absence of gastrointestinal symptoms. HLA typing was not done. The patient was treated with ibuprofen (200 - 400 mg orally three times daily) and physical therapy. After initial improvement, she presented six weeks later with worsening pain, redness and swelling of the left first metatarsophalangeal joint. Intra-articular injection of 6 mg triamcinolone-hexacetonide led to resolution of symptoms. The patient discontinued taking the ibuprofen one month later. When seen three months after the injection, she was without complaints and had a normal musculoskeletal exam. At 18 months follow up she continues to be free of symptoms.

Post-viral arthritis can occur immediately after the infection or several weeks or months later [5,6] and typically affects joints of the lower extremity, most commonly knees and ankles [6]. Although it is impossible to prove causality in this single case, we would like to suggest that this patient's arthritis represents a rare case of post-infectious ("reactive") arthritis after influenza B virus infection. This notion is supported by the following observations: (1) influenza B virus was demonstrated by two different assays two weeks prior to onset of rheumatologic manifestations, (2) the clinical course was relatively benign and short, and (3) the affected joints are uncommonly involved in the oligoarticular form of juvenile idiopathic arthritis [7], which would be an alternate likely diagnosis in this patient.

What might be the mechanism of this post-viral arthritis? Thus far, influenza-associated synovitis has only been described after influenza immunization [2-4], but some mechanistic insights may be gleaned from this observation. The systemic response to challenge with a new antigen often involves the synovium, as T cells reactive against the respective antigens have been detected in synovial membranes of adult patients with rheumatoid arthritis after immunization with tetanus or influenza vaccines [8]. Normal synovium, too, contains low levels of T cells and macrophages [9] and dendritic cells, and it is possible that the antigenic challenge of an acute viral infection leads to a further influx of inflammatory cells, which could potentially lead to synovitis in a previously healthy joint. Molecular mimicry (due to similarities between synovial and viral antigenic epitopes) and synthesis of virus-induced cytokines that trigger autoimmune-mediated tissue injury are other proposed mechanisms of post-viral arthritis [2,10]. However, clinical flares of RA after influenza infection or immunization, or new synovitis after influenza immunization, occur rarely, indicating that host factors, and perhaps other environmental factors, play important roles.

## Conclusions

This report represents the first detailed description of a possible post-infectious arthritis following infection with an influenza virus. Naturally, it is impossible to prove causality in this single case. Thus, this report merely intends to raise awareness among clinicians to consider a recent influenza virus infection in the differential diagnosis of new-onset arthritis in children and adolescents, and perhaps also in adults, and to generate interest in formulating prospective studies on influenza viruses causing peri- or post-infectious arthritis.

## Competing interests

The authors declare that they have no competing interests.

## Authors' contributions

NB and FP cared for the patient and wrote the manuscript. MG participated in the care of the patient, provided helpful discussion, and read the manuscript.

## Consent

Written consent to publish this case report was obtained from the patient and her mother. A copy of the signed consent form is available for review by the editor of this journal.
